# Rhythm control without catheter ablation may have benefits beyond stroke prevention in rivaroxaban-treated non-permanent atrial fibrillation

**DOI:** 10.1038/s41598-022-07466-z

**Published:** 2022-03-08

**Authors:** Wei-Ru Chiou, Po-Lin Lin, Chun-Che Huang, Jen-Yu Chuang, Lawrence Yu-Min Liu, Min-I Su, Feng-Ching Liao, Jen-Yuan Kuo, Cheng-Ting Tsai, Yih-Jer Wu, Kuang-Te Wang, Ying-Hsiang Lee

**Affiliations:** 1 Division of Cardiology, Taitung MacKay Memorial Hospital, Taitung, Taiwan; 2 Department of Medicine, MacKay Medical College, New Taipei, Taiwan; 3 Division of Cardiology, Hsinchu MacKay Memorial Hospital, Hsinchu, Taiwan; 4 Department of Biological Science and Technology, National Yang Ming Chiao Tung University, Hsinchu, Taiwan; 5 Department of Healthcare Administration, I-Shou University, Kaohsiung, Taiwan; 6 Department of Medical Education, MacKay Memorial Hospital, Taipei, Taiwan; 7 Cardiovascular Center, MacKay Memorial Hospital, 92, Zhongshan North Road Section 2, Zhongshan District, Taipei, Taiwan; 8 Department of Artificial Intelligence and Medical Application, MacKay Junior College of Medicine, Nursing, and Management, Taipei, Taiwan

**Keywords:** Cardiology, Cardiovascular biology, Cardiovascular diseases, Arrhythmias, Atrial fibrillation

## Abstract

The current treatment paradigm for atrial fibrillation (AF) prioritizes rate control over rhythm control; however, rhythm control has shown benefits over other AF strategies. This study compares the outcomes of rivaroxaban with and without concomitant antiarrhythmic drugs (AADs), using propensity score matching to correct for statistical effects of baseline discrepancies. This multi-center retrospective study included 1,477 patients with non-permanent AF who took rivaroxaban for at least one month between 2011 and 2016 and had not received catheter ablation. Concomitant AAD use was compared against clinical outcome endpoints for effectiveness, safety, and major adverse cardiac events (MACE). Associations with concomitant AAD use were evaluated using multivariate Cox proportional hazard analyses. Patients were divided into two matched groups: rivaroxaban alone (n = 739) and with concomitant AADs (n = 738). The cumulative incidences of safety (p = 0.308), effectiveness (p = 0.583), and MACE (p = 0.754) were similar between the two groups, and multivariate analysis showed no significant differences. The new thromboembolism and all-cause death rates were higher in rivaroxaban alone (2.7% vs 0.8%, p = 0.005; and 10% vs. 6.9%, p = 0.032, respectively). The heart failure readmission rate was higher in the concomitant-AAD group (8.4% vs. 13.3%, p = 0.003). The concomitant use of rivaroxaban with AADs appears to be well-tolerated, with lower rates of thromboembolism and all-cause death, but is associated with more occurrences of congestive heart failure.

## Introduction

The current treatment paradigm for atrial fibrillation (AF) prioritizes rate control over rhythm control, a direct result of large controlled trials, such as the AFFIRM trial from 2002, which concluded that, with regards to AF, there were no survival advantages to rhythm control over rate control and have further suggested that there may be advantages of rate control over rhythm control, such as lower rates of hospitalization and adverse drug effects^1^. However, rhythm control using dronedarone can reduce cardiovascular hospitalization or death and may be beneficial for stroke prevention, according to the ATHENA study and associated post-hoc analysis^2^. The EAST AFNET 4 trial investigators found that, compared with usual care, early initiation of rhythm-control therapy was associated with less-frequent cardiovascular events^3^. This early rhythm-control therapy included antiarrhythmic drugs, atrial fibrillation ablation, as well as cardioversion of persistent atrial fibrillation which were administered concomitantly with anticoagulants^3^. However, according the AFFIRM and ANDROMEDA trials, the primary potential disadvantage of rhythm-control strategies was a higher risk of adverse drug effects, especially for patients with congestive heart failure (CHF)^1,4^. It is still unknown whether rhythm control has any particular advantages over other AF strategies, especially without catheter ablation. As such, the interactions between rhythm-control and other drugs are extremely relevant.

Oral anticoagulants are the primary management method for the risk of ischemic stroke due to AF. Non-Vitamin-K antagonists (NOACs) such as rivaroxaban have increasingly replaced Vitamin-K antagonists (warfarin) due to better safety profiles and less risk of intercranial damage^5,6^. Antiarrhythmic drugs (AADs), such as amiodarone, propafenone, and relative newcomer dronedarone are often also prescribed to patients with non-permanent AF to maintain sinus rhythm. There is a need for data regarding concomitant use of NOACs and AADs, given their high levels of co-administration in clinical practice^7^.

Previously, Chiou et al*.* showed in a multicenter, retrospective cohort study that the concomitant use of rivaroxaban with AADs, including dronedarone, seemed to be well tolerated. However, concomitant amiodarone showed significantly higher lower left ventricular fraction rates (LVEF), possibly due to higher rates of heart failure in that particular patient population prior to the study^8^.

The present study was designed to compare the effectiveness and safety between rivaroxaban alone and with concomitant AADs, using propensity score matching of underlying characteristics such as LVEF to correct for the statistical effect of baseline discrepancies.

## Patients and methods

### Data source and study population

This retrospective cohort study involved the electronic medical records of four different MacKay Memorial Hospitals in four Taiwanese cities. Study protocol IRB No. 16MMHIS009 was approved by the Institutional Review Board of MacKay Memorial Hospital, which waived the requirement for informed consent, as this was a retrospective study. This study complied with the Declaration of Helsinki.

**Figure 1:**
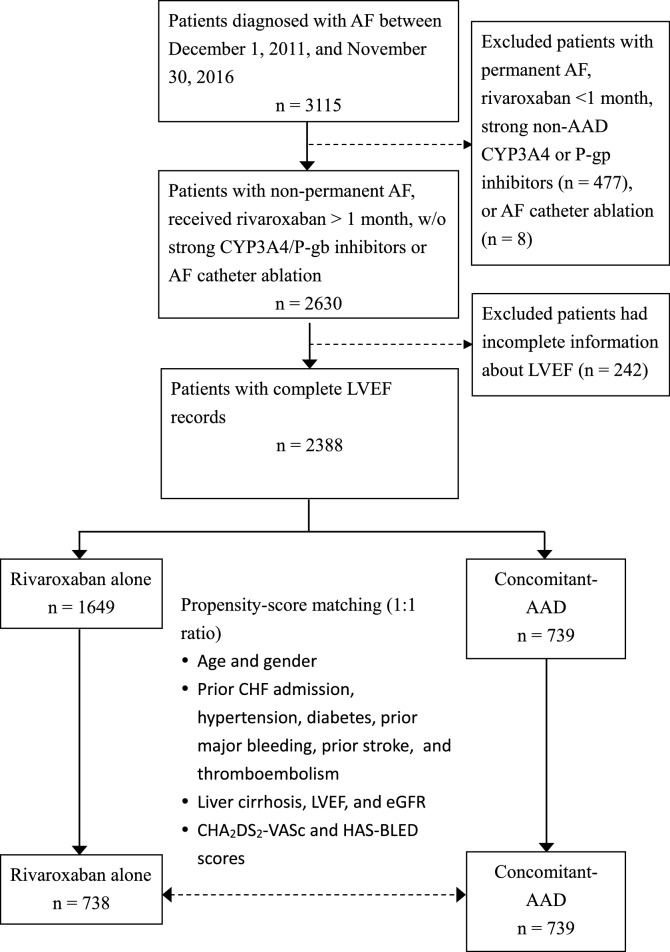
Flow diagram of patient selection. AADs, antiarrhythmic drugs; AF, atrial fibrillation; CHF, congestive heart failure; eGFR, estimated glomerular filtration rate; LVEF, left ventricular ejection fraction.

The patient selection process is presented in Fig. 1. Patients were enrolled on the criteria of having received a diagnosis of AF between December 1, 2011, and November 30, 2016. Patients were excluded based on having permanent AF, receiving rivaroxaban for less than a month, being prescribed strong non-AAD CYP3A4 or permeability-glycoprotein (P-gp) inhibitors (rifampicin, HIV protease inhibitors, itraconazole, ketoconazole and voriconazole), or having received AF catheter ablation. Cases with incomplete information about LVEF were also excluded.

The remaining patients were divided into two groups according to medication: rivaroxaban alone vs. rivaroxaban plus amiodarone, propafenone, and dronedarone (“concomitant-AAD”) for at least 28 days. Propensity score matching was applied to match patients from both groups in a 1:1 ratio. The follow-up period was from treatment initiation until discontinuation or the end of the study (31 December 2017). Patients who received rivaroxaban alone were the control group.

### Clinical outcomes: safety and effectiveness endpoints and MACE

Independent healthcare professionals reviewed the patients’ electronic medical records to determine if the safety and effectiveness endpoints were met or whether a major adverse cardiac event (MACE) had occurred.

The safety endpoint used the criteria set forth by the International Society on Thrombosis and Haemostasis (ISTH) and comprised major bleeding, critical site bleeding, minor bleeding, and fatal bleeding. Major bleeding was defined as a Hb fall of ≥ 2 g/dL or needing a transfusion of ≥ 2 U PRBC; critical site bleeding occurred in a critical area or organ, such intracranial, intraocular, intraspinal, intra‐articular, pericardial or retroperitoneal bleeding, or intramuscular bleeding accompanied by compartment syndrome; and minor bleeding was any sign or symptom of hemorrhage that, although not meeting the ISTH criteria for major bleeding, still led to increased care or hospitalization, medical intervention by a healthcare professional, or at least a face to face evaluation^9^. The effectiveness endpoint was comprised of stroke (ischemic stroke and hemorrhagic stroke) and systemic thromboembolism (any thromboembolism event other than ischemic stroke). Any combination of hemorrhagic stroke, ischemic stroke, systemic thromboembolism, myocardial infarction, or cardiovascular death was considered a MACE.

### Covariates

The following covariates were included: age, gender, prior congestive heart failure (CHF) admission, hypertension, diabetes, prior major bleeding, stroke, transient ischemic attack (TIA), systemic thromboembolism, liver cirrhosis, LVEF, estimated glomerular filtration rate (eGFR), as well as other medications (including inhibitors of renin-angiotensin system, beta-blocker, statin, aspirin, clopidogrel or ticagrelor, and nonsteroidal anti-inflammatory drug). In addition, the risk scores (HAS-BLED) for bleeding and stroke (CHA_2_DS_2_-VASc) were calculated according to the information at baseline.

### Statistical methods

SPSS (version 24) software (IBM Corp, New York, USA) was used for all statistical analyses. To reduce imbalanced covariates, a propensity score matching method was applied to match patients from rivaroxaban alone and rivaroxaban plus concomitant AADs groups in a 1:1 ratio (Fig. 1). Distribution of patient demographics, preexisting comorbidities, LVEF, eGFR, other medications, as well as effectiveness, safety, and MACE endpoints between the two groups were examined using Fisher’s exact test for categorical variables, and student's t test for continuous variables, respectively. The cumulative cause-specific incidences of safety, effectiveness, and MACE within each of the two patient groups was estimated using the Aalen-Johansen estimator, and the Gray test was performed in order to compare the cumulative incidence function of primary outcomes between the two different patient groups. The influence of the use of rivaroxaban with and without concomitant AADs on the effectiveness, safety, and MACE endpoints was estimated using the multivariate Cox proportional hazards model with the robust sandwich variance estimator. Hazard ratios (HR) and 95% confidence intervals (CI) were calculated, and a p-value < 0.05 was considered statistically significant.

### Ethial approval

Study protocol IRB No. 16MMHIS009 was approved by the Institutional Review Board of MacKay Memorial Hospital, which waived the requirement for informed consent, as this was a retrospective study.

## Results

### Baselines and covariates

A total of 3115 patients were enrolled into the study. 477 patients were excluded based on permanent AF, rivaroxaban durations of less than a month, and the use of rifampicin, HIV protease inhibitors, itraconazole, ketoconazole or voriconazole. 8 patients who received AF catheter ablation also excluded, as well as 242 cases that had incomplete LVEF information. From the remainder, a total of 739 patients with receiving rivaroxaban plus AADs for more than 28 days (the concomitant-AAD group) were identified. From the 1649 patients who received rivaroxaban alone, 738 matched patients were identified (the rivaroxaban alone group) (Fig. 1). During the study period, the persistence rate with the same AAD was 76.2% (563 patients) in the concomitant-AAD group.

Table 1 compares the patient and provider characteristics of the concomitant-AAD and rivaroxaban alone groups before and after propensity score matching. Before propensity score matching, the prevalence of hypertension (p = 0.003) was numerically higher in the concomitant-AAD group. On the other hand, the incidence of prior CHF admission (p < 0.001), major bleeding (p = 0.022), stroke/TIA/systemic thromboembolism (p < 0.001) as well as isolated prior stroke/TIA (p < 0.001) were significantly higher in the rivaroxaban alone group. The rivaroxaban alone group also had more impaired renal function with a significantly lower eGFR (p < 0.001) compared with the concomitant-AAD group. The concomitant medications in both groups before propensity score matching showed no statistically significant differences. 606 (82.0%) patients in the concomitant-AAD group and 601 (81.4%) patients in the matched rivaroxaban alone group had paroxysmal atrial fibrillation and showed no statistically significant differences (p = 0.788).

**Table 1:** Baseline characteristics of atrial fibrillation patients receiving rivaroxaban with and without anti-arrhythmic drugs before and after propensity score matching.

Characteristics	Concomitant-AAD (n = 739)		Rivaroxaban alone Unmatched (n = 1649)		*P*	Rivaroxaban alone Matched (n = 738)		*P*
Age, yr, mean (SD)	74.1	(10.1)	73.3	(12.3)	0.084	74.3	(11.1)	0.737
Female, no. (%)	358	(48.4)	829	(50.3)	0.426	374	(50.7)	0.405
AF type					0.109			0.788
Paroxysmal AF, no. (%)	606	(82.0)	1304	(79.1)		601	(81.4)	
Persistent AF, no. (%)	133	(18.0)	345	(20.9)		137	(18.6)	
Pre-CHF admission, no. (%)	216	(29.2)	622	(37.7)	< 0.001	223	(30.2)	0.690
Hypertension, no. (%)	594	(80.4)	1235	(74.9)	0.003	597	(80.9)	0.843
Diabetes, no. (%)	256	(34.6)	552	(33.5)	0.575	260	(35.2)	0.827
Prior major bleeding, no. (%)	49	(6.6)	157	(9.5)	0.022	33	(4.5)	0.088
Prior TIA/stroke, no. (%)	130	(17.6)	419	(25.4)	< 0.001	131	(17.7)	0.946
Prior TIA/ Stroke/Systemic thromboembolism, no. (%)	150	(20.3)	499	(30.3)	< 0.001	151	(20.5)	0.949
Liver cirrhosis, no. (%)	28	(3.8)	84	(5.1)	0.175	31	(4.2)	0.693
LVEF (%), mean (SD)	60.1	(10.3)	60.0	(9.8)	0.778	60.0	(9.9)	0.791
eGFR, mL/mm/1.73m^2^, mean (SD)	64.3	(23.0)	70.2	(28.4)	< 0.001	66.1	(27.5)	0.171
Medication								
ACEI / ARB, no. (%)	455	(61.6)	1019	(61.8)	0.927	473	(64.1)	0.333
Beta-blocker, no. (%)	234	(31.7)	548	(33.2)	0.479	265	(35.9)	0.088
Statin, no. (%)	240	(32.5)	529	(32.1)	0.850	233	(31.6)	0.738
Aspirin, no. (%)	34	(4.6)	66	(4.0)	0.508	31	(4.2)	0.800
Clopidogrel or brilinta, no. (%)	28	(3.8)	64	(3.9)	1.000	24	(3.2)	0.672
NSAID, no. (%)	97	(13.1)	224	(13.6)	0.795	89	(12.1)	0.583

### Propensity score-matched analysis

The total number of patients included in the analysis was 1,477 (738 in the rivaroxaban alone group, 739 in the concomitant-AAD group). After matching for propensity score, no significant differences were identified among the covariates, including prior CHF admission, hypertension, diabetes, renal function, major bleeding and any brain or systemic thromboembolic events. Table 2 compares bleeding and clinical events between the matched groups. Between the two groups, there were no statistically significant differences in the safety and effectiveness endpoints and the incidence of MACE. 11.6% of the patients in the rivaroxaban alone group and 13.5% in the concomitant-AAD group met the safety endpoint (p = 0.308). 3.9% of patients in the rivaroxaban alone group and 3.4% of the concomitant-AAD group met the effectiveness endpoint (p = 0.583). The incidence of MACE in the rivaroxaban alone group was 6.8% and, in the concomitant-AAD group, was 6.4% (p = 0.754).

**Table 2:** Bleeding and clinical events of atrial fibrillation patients receiving rivaroxaban with and without anti-arrhythmic drugs in the propensity score-matched analysis.

	Rivaroxaban alone (n = 738)		Concomitant-AAD (n = 739)		P
Follow-up time (months), mean (SD)	31.4	(10.8)	31.9	(10.7)	0.413
Safety endpoint, no. (%)	86	(11.6)	100	(13.5)	0.308
Bleeding needs transfusion ≥ 2U or Hb drop ≥ 2 g/dL, no. (%)	21	(2.8)	23	(3.1)	0.879
GI bleeding, no. (%)	31	(4.2)	31	(4.2)	1.000
Effectiveness endpoint, no. (%)	29	(3.9)	25	(3.4)	0.583
New ischemic stroke, no. (%)	9	(1.2)	13	(1.8)	0.520
New hemorrhagic stroke, no. (%)	2	(0.3)	6	(0.8)	0.288
New stroke (new ischemic stroke and hemorrhagic stroke), no. (%)	10	(1.4)	18	(2.4)	0.181
Systemic thromboembolism, no. (%)	20	(2.7)	6	(0.8)	0.005
MACE, no. (%)	50	(6.8)	47	(6.4)	0.754
Non-fatal MI, no. (%)	7	(0.9)	12	(1.6)	0.356
HF readmission, no. (%)	62	(8.4)	98	(13.3)	0.003
With AF relapse, no. (%)	15	(2.0)	31	(4.1)	0.024
Without AF relapse, no. (%)	47	(6.4)	67	(9.2)	0.064
ARF, no. (%)	7	(0.9)	5	(0.7)	0.579
CV death, no. (%)	19	(2.6)	12	(1.6)	0.210
All cause death, no. (%)	74	(10.0)	51	(6.9)	0.032

Effectiveness endpoint: new ischemic stroke, ICH, or embolism. Safety endpoint (by ISTH definition): Hb fall ≥ 2 g/dL or transfusion ≥ 2 U PRBC, critical site bleeding, or fatal bleeding.

AF, Atrial fibrillation; MACE, CV death; MI, new ischemic stroke, embolism, or ICH.

In addition, the incidence levels between the two groups were also similar for new ischemic stroke (p = 0.520), new hemorrhagic stroke (p = 0.288), bleeding requiring transfusion greater than 2 units or hemoglobin drops greater than 2 g/dL (p = 0.879), gastrointestinal bleeding (p = 1.000) and non-fatal myocardial infraction (p = 0.356).

**Figure 2:**
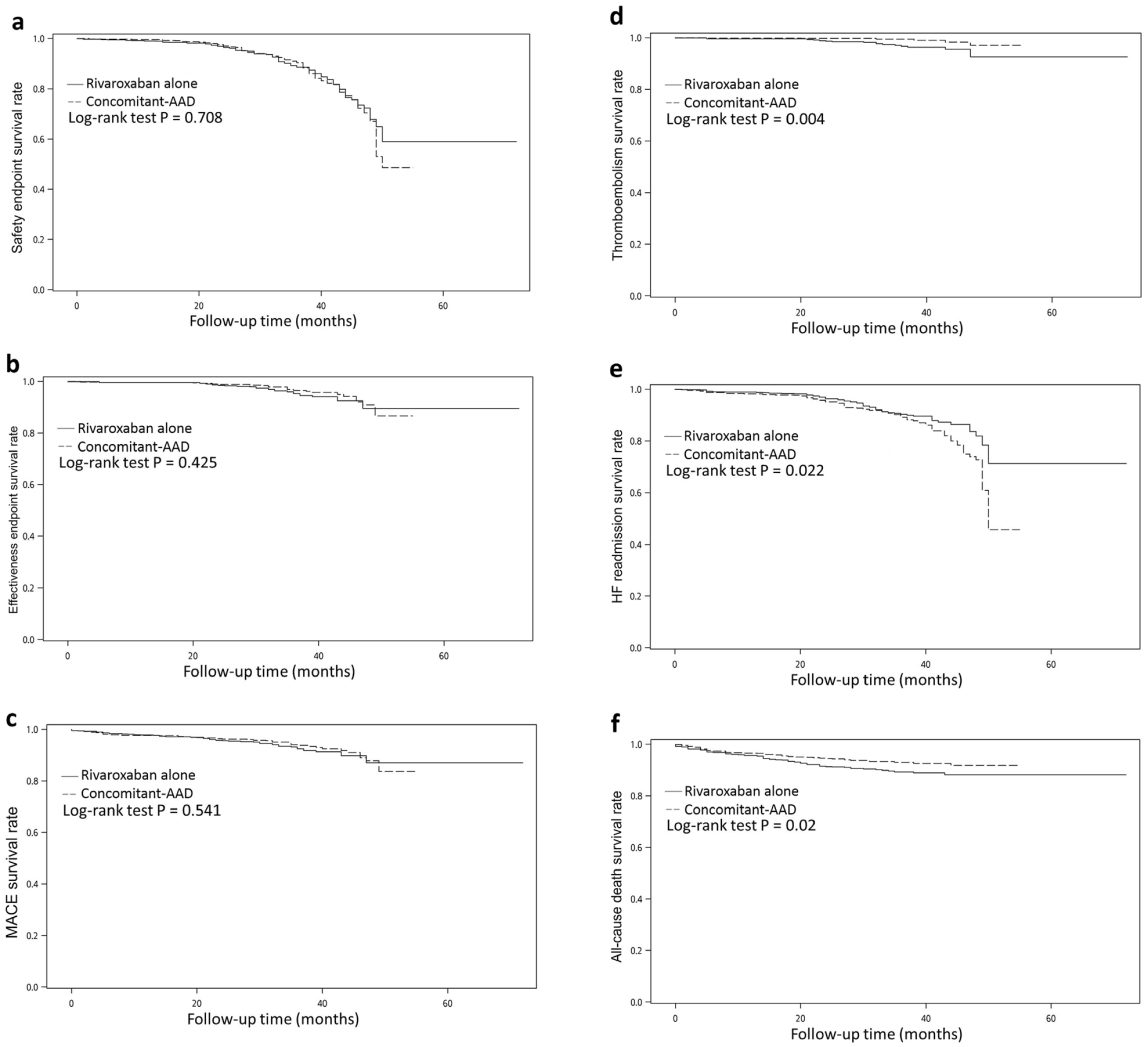
Survival rates for: (**a**) safety endpoint (composite of major bleeding and minor bleeding); (b) effectiveness endpoint (composite of stroke and systemic thromboembolism); (**c**) MACE; (**d**) thromboembolism; (**e**) HF readmission; (**f**) all-cause death. *MACE* major adverse cardiac events, *HF* heart failure

The occurrence rate of new thromboembolisms was slightly higher in the rivaroxaban alone group than in the concomitant-AAD group: 2.7% vs 0.8%, respectively, with p = 0.005. Three cases of new thromboembolisms occurred during AF relapse, and the rates were not significantly different between the groups (rivaroxaban alone 0.3% vs. concomitant-AAD 0.1%, p = 0.624). Likewise, the all-cause death rate was also higher in the rivaroxaban alone group: 10% vs. 6.9%, p = 0.032. On the contrary, the HF-readmission rate was higher in the concomitant-AAD group: 8.4% vs. 0.3%, p = 0.003 (Fig. 2). In the concomitant-AAD group, the increase of HF readmission rate with AF replace was statistically significant (rivaroxaban alone 2.0% vs. concomitant-AAD 4.1%, p = 0.024) and modest without AF relapse (rivaroxaban alone 6.4% vs. concomitant-AAD 9.2%, p = 0.064) (Table 2).

**Figure 3:**
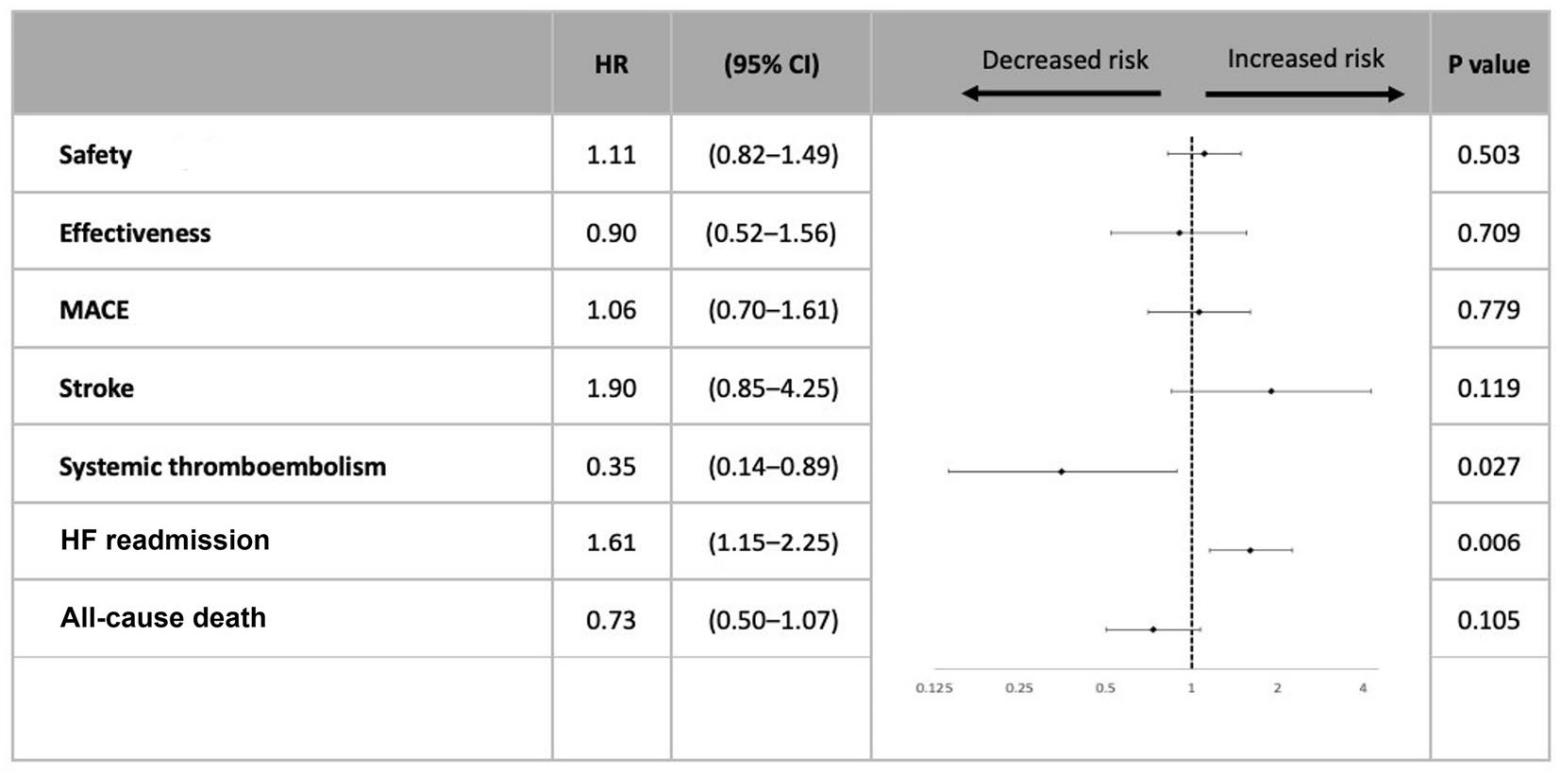
Comparing of multivariate Cox regression analysis for endpoints in patients with atrial fibrillation receiving rivaroxaban with concomitant AADs.

Using multivariate Cox regression analysis, no statistically significant differences were found between the two groups regarding the safety endpoint (HR 1.11 [95% CI: 0.82‒1.49], p = 0.503), effectiveness endpoint (HR 0.90 [95% CI: 0.52‒1.56], p = 0.709) and MACE (HR 1.06 [95% CI 0.70‒1.61], p = 0.779) (Table 3). In the concomitant-AAD group, the HR of systemic thromboembolism (HR 0.35 [95% CI 0.14‒0.89], p = 0.027) was numerically lower, but the HF readmission rate (HR 1.61 [95% CI 0.15‒2.25], p = 0.006) was higher (Fig. 3). There was no difference in all-cause death (HR 0.73 [95% CI 0.50‒1.07], p = 0.105) after multivariate Cox regression analysis (Supplementary Table S1).

**Table 3:** Multivariate Cox regression analysis for effectiveness, safety, and MACE outcomes of atrial fibrillation patients with receiving either rivaroxaban alone or concomitant AADs.

	Effectiveness endpoint			Safety endpoint			MACE		
	HR	(95% CI)	P value	HR	(95% CI)	P value	HR	(95% CI)	P value
Concomitant-AAD class									
Rivaroxaban alone	1.00			1.00			1.00		
Rivaroxaban plus AADs	0.90	(0.52‒1.56)	0.709	1.11	(0.82‒1.49)	0.503	1.06	(0.70‒1.61)	0.779
Age (yr)	1.01	(0.98‒1.05)	0.369	1.01	(0.99‒1.02)	0.414	1.02	(1.00‒1.05)	0.080
Female	0.81	(0.45‒1.46)	0.487	1.12	(0.82‒1.52)	0.477	0.98	(0.62‒1.54)	0.924
Pre-CHF admission	0.76	(0.38‒1.52)	0.440	1.02	(0.72‒1.45)	0.894	0.89	(0.54‒1.47)	0.655
Hypertension	1.51	(0.65‒3.53)	0.341	1.10	(0.73‒1.65)	0.659	1.47	(0.77‒2.80)	0.243
Diabetes	0.87	(0.48‒1.60)	0.659	1.16	(0.85‒1.59)	0.345	1.01	(0.64‒1.61)	0.954
Prior major bleeding	0.95	(0.27‒3.33)	0.936	2.56	(1.64‒4.01)	< 0.001	0.35	(0.10‒1.21)	0.096
Prior TIA/stroke	0.37	(0.14‒0.98)	0.046	1.17	(0.46‒2.99)	0.747	0.42	(0.19‒0.94)	0.036
Prior TIA/stroke Thromboembolism	5.52	(2.30‒13.28)	< 0.001	0.75	(0.31‒1.86)	0.539	3.03	(1.49‒6.16)	0.002
Liver cirrhosis	0.59	(0.13‒2.75)	0.504	1.13	(0.58‒2.18)	0.724	1.08	(0.42‒2.77)	0.875
LVEF (%)	0.98	(0.95‒1.01)	0.252	0.99	(0.97‒1.01)	0.177	0.99	(0.97‒1.02)	0.524
eGFR (mL/mm/1.73m^2^)	1.00	(0.99‒1.01)	0.425	1.00	(0.99‒1.01)	0.455	1.00	(0.99‒1.01)	0.990
Medication									
ACEI/ ARB	0.79	(0.43‒1.46)	0.455	0.96	(0.69‒1.34)	0.817	0.80	(0.49‒1.28)	0.348
Beta-blocker	0.79	(0.43‒1.46)	0.455	1.01	(0.73‒1.38)	0.977	0.75	(0.47‒1.21)	0.241
Statin	1.63	(0.91‒2.92)	0.104	0.76	(0.53‒1.07)	0.116	1.34	(0.83‒2.17)	0.229
Aspirin	1.20	(0.30‒4.71)	0.798	0.49	(0.17‒1.42)	0.188	1.01	(0.41‒2.54)	0.976
Clopidogrel or brilinta	1.31	(0.41‒4.23)	0.653	0.87	(0.34‒2.23)	0.778	1.88	(0.84‒4.22)	0.124
NSAID	1.39	(0.58‒3.35)	0.459	0.98	(0.59‒1.63)	0.945	1.47	(0.76‒2.85)	0.254

AADs, anti-arrhythmia drugs.

Older patients had higher rate of all-cause death (HR 1.02 [95% CI 1.00‒1.05], p = 0.022), and patients with prior TIA/stroke had significantly lower risks of meeting the effectiveness endpoint (HR 0.37 [95% CI 0.14‒0.98], p = 0.046) and MACE (HR 0.42 [95% CI 0.19‒0.94], p = 0.036). Patients with prior TIA/stroke and systemic thromboembolism had significantly higher levels of risk, which met the effectiveness endpoint (HR 5.52 [95% CI 2.30‒13.28], p < 0.001), MACE (HR 3.03 [95% CI 1.49‒6.16], p = 0.002) and more systemic thromboembolisms (HR 4.14 [95% CI 1.07‒16.02], p = 0.040). Prior major bleeding was associated with significantly higher risks of meeting safety endpoint (HR 2.56 [95% CI 1.64‒4.01], p < 0.001) (Table 3), and prior CHF admission was related to higher HF readmission rate in the concomitant-AAD group (HR 2.48 [95% CI 1.72‒3.59], p < 0.001) (Supplementary Table S1).

## Discussion

The present study assesses the real-world outcomes of rivaroxaban with and without concomitant AADs and excluded AF catheter ablation. Currently, the treatment paradigm for AF prioritizes rate control over rhythm control, due to large controlled studies like AFFIRM that concluded that there were no survival advantages to rhythm control over rate control^1^. However, in the EAST AFNET 4 study of 2,789 patients across 135 centers, the early administration of AADs, ablation, and/or cardioversion was associated with fewer cardiovascular events than just the usual care^3^. AFFIRM revealed that there may be advantages of rate control over rhythm control, but it was conducted with warfarin as the main anticoagulant, which has been largely replaced by NOACs such as rivaroxaban^10^, which was the predominant NOAC in our hospital and in Taiwan. In addition, a new AAD, dronedarone, was approved and released after the AFFIRM study. Dronedarone shows fewer adverse effects than amiodarone^11^, may lead to better cardiovascular outcomes(1), and could reduce the incidence of stroke when compared with earlier AADs^12^. It has been observed that patients who are given dronedarone or flecainide for rhythm control in AF have lower rates of comorbidity^13^.

According the current 2020 European Society of Cardiology (ESC) Guidelines for the diagnosis and management of atrial fibrillation, the indication for rhythm control is to reduce AF-related symptoms and improve quality of life, and there is no substantial evidence for any different outcome by rhythm control strategy^14^. However, subsequent investigators have considered the possibility that rhythm control drugs should be started in the early stages of AF in order to achieve better responses, as lower baseline AF burdens were associated with greater relative reductions by AADs^15^.

In a retrospective cohort study on a Taiwanese population with non-valvular AF, there was no significant difference in the adjusted incidence rate ratio of major bleeding risk between rivaroxaban alone and rivaroxaban with concomitant dronedarone (0.92, 99% CI 0.68–1.24)^16^. Concomitant use of rivaroxaban with AADs, including dronedarone, appeared to be well-tolerated in one multicenter, retrospective cohort study^8^. Our study illustrates rhythm control strategies used in Taiwan from 2011 to 2016 and included two post-AFFIRM drugs – dronedarone, an AAD, combined with rivaroxaban, an anti-coagulant – both without catheter ablation. It highlights not only their combined safety but also shows that they are potentially beneficial with regards to thromboembolism and all-cause death.

The data support that there were no significant differences in safety issues when rivaroxaban was taken alone versus in combination with AADs (p = 0.803). Both groups achieved the same effectiveness endpoint during the follow-up period, but systemic thromboembolism rates were significantly lower in the group with concomitant AADs. The thromboembolic events were not correlated with AF relapse, similar to Brambatti et al.’s study of patients with implantable pacemakers and defibrillators^17^. These data suggest that not only could combining NOACs with rhythm control by AADs be a feasible therapeutic approach but that it could be one more beneficial than anticoagulant therapy alone.

The patients with prior TIA/stroke without thromboembolism were significantly less likely to meet effectiveness endpoint and/or experience MACE, suggesting that rivaroxaban provided an additional benefit for such patients. However, the patients with prior TIA/stroke with thromboembolism were more likely to meet the effectiveness endpoint or develop MACE and/or new thromboembolism. Perhaps prior thromboembolism poses another independent factor for effectiveness endpoint and MACE, due to other comorbidities, such as immobility, being bed-ridden, or autoimmune diseases that could lead to coagulopathy, like antiphospholipid syndrome.

Two independent factors correlated with the all-cause death rate: age and angiotensin-converting enzyme inhibitor (ACEI)/angiotensin receptor blocker (ARB) use. Older patients showed a higher mortality rate (p = 0.022), and patients taking ACEI/ ARB showed a lower mortality rate (p = 0.044). Prior major bleeding seriously affected the safety endpoint (p < 0.001).

In our multivariate Cox regression analysis, patients in the concomitant-AAD group with previous HF admission history were associated with a higher HF readmission rate, which could be due to the use of propafenone or dronedarone as rhythm control medications. The rates of HF readmission with and without AF relapse were both higher in the concomitant-AAD group than in the rivaroxaban alone group. Catheter ablation for atrial fibrillation has been proposed as a means of improving outcomes among patients with heart failure^18,19^. A recent study also supported the idea that catheter ablation for atrial fibrillation in patients with heart failure was associated with a significantly lower rate of all-cause death or re-hospitalization for worsening heart failure than medical therapy^20^ and that catheter ablation could also result in greater improvement in LVEF, quality of life and functional status in these patients^21^.

One limitation of the present study was an insufficiently large patient population. Another limitation was that further analysis showed that prior systemic thromboembolism could be potentially independently classified as an underlying cause and should therefore be investigated for its impact on the effectiveness endpoint and MACE. A third limitation was that although patients receiving non-AAD strong CYP3A4 or P-gp inhibitors were excluded – specifically HIV protease inhibitors, itraconazole, ketoconazole, voriconazole, and rifampicin – we could not exclude all such drugs from our study. Therefore, the possibility that the concomitant administration of such medications with rivaroxaban could have drug interactions cannot be ruled out, although the 2018 EHRA guidelines suggest that such interactions should be limited, if they do exist^22^. The fourth limitation was that the persistence rate with the same AAD was 76.2% in the concomitant-AAD group. However, AAD switching is common in patients who are followed for a long time, which may affect clinical outcomes. Like the EAST AFNET 4 trial, the persistence rate was 70.3% in the rhythm control group but still showed a benefit^3^. Lastly, we did not evaluate if our patients had ever been prescribed AADs before rivaroxaban initiation and thus did not include the influence of ADD use before rivaroxaban initiation in this study.

## Conclusion

This real-world study demonstrated fewer systemic thromboembolism and all-cause death and more CHF admission in patients who used concomitant AADs with rivaroxaban. After multivariate adjustment, the use of concomitant AADs was independently associated with fewer occurrences of thromboembolism and more occurrences of CHF readmission. These findings warrant further investigation for the safety and efficacy of rhythm control without catheter ablation.

## Supplementary Information

Dataset S1.

Dataset S2.

Supplementary Table S1.

## Data Availability

The data underlying this article are available in the article and in its online supplementary material.

## References

[1] Wyse DG, Waldo AL, DiMarco JP (2002). A comparison of rate control and rhythm control in patients with atrial fibrillation. N. Engl. J. Med..

[2] Hohnloser SH, Crijns HJGM, van Eickels M (2009). Effect of dronedarone on cardiovascular events in atrial fibrillation. N. Engl. J. Med..

[3] Kirchhof P, Camm AJ, Goette A (2020). Early rhythm-control therapy in patients with atrial fibrillation. N. Engl. J. Med..

[4] Køber L, Torp-Pedersen C, McMurray JJV (2008). Increased mortality after dronedarone therapy for severe heart failure. N. Engl. J. Med..

[5] Patel MR, Mahaffey KW, Garg J (2011). Rivaroxaban versus warfarin in nonvalvular atrial fibrillation. N. Engl. J. Med..

[6] Hori M, Matsumoto M, Tanahashi N (2012). Rivaroxaban vs. warfarin in Japanese patients with atrial fibrillation—The J-ROCKET AF study. Circ. J..

[7] Celikyurt I, Meier CR, Kühne M, Schaer B (2017). Safety and interactions of direct oral anticoagulants with antiarrhythmic drugs. Drug Saf..

[8] Chiou W-R, Huang C-C, Lin P-L (2021). Safety and effectiveness of rivaroxaban in combination with various antiarrhythmic drugs in patients with non-permanent atrial fibrillation. Am. J. Cardiovasc. Drugs.

[9] Kaatz S, Ahmad D, Spyropoulos AC, Schulman S (2015). Definition of clinically relevant non-major bleeding in studies of anticoagulants in atrial fibrillation and venous thromboembolic disease in non-surgical patients: communication from the SSC of the ISTH. J. Thromb. Haemost..

[10] Chan Y-H, See L-C, Tu H-T (2018). Efficacy and Safety of Apixaban, Dabigatran, Rivaroxaban, and Warfarin in Asians With Nonvalvular Atrial Fibrillation. J. Am. Heart Assoc..

[11] Santangeli P, Di Biase L, Pelargonio G, Burkhardt JD, Natale A (2011). The pharmaceutical pipeline for atrial fibrillation. Ann. Med..

[12] Freemantle N, Lafuente-Lafuente C, Mitchell S, Eckert L, Reynolds M (2011). Mixed treatment comparison of dronedarone, amiodarone, sotalol, flecainide, and propafenone, for the management of atrial fibrillation. Europace.

[13] Friberg L (2018). Safety of apixaban in combination with dronedarone in patients with atrial fibrillation. Int. J. Cardiol..

[14] Hindricks G, Potpara T, Dagres N (2021). 2020 ESC Guidelines for the diagnosis and management of atrial fibrillation developed in collaboration with the European Association for Cardio-Thoracic Surgery (EACTS): The Task Force for the diagnosis and management of atrial fibrillation of the Europe. Eur. Heart J..

[15] Lin P-L, Huang C-C, Wu Y-J (2019). Relations between baseline burden, maximum duration, and relative reduction of atrial fibrillation: Insights from continuous monitoring in rhythm control. J. Cardiovasc. Electrophysiol..

[16] Chang S-H, Chou I-J, Yeh Y-H (2017). Association between use of non-vitamin K Oral anticoagulants with and without concurrent medications and risk of major bleeding in nonvalvular atrial fibrillation. JAMA.

[17] Brambatti M (2014). Temporal relationship between subclinical atrial fibrillation and embolic events. Circulation.

[18] Wazni OM, Marrouche NF, Martin DO (2005). Radiofrequency ablation vs antiarrhythmic drugs as first-line treatment of symptomatic atrial fibrillation: a randomized trial. JAMA.

[19] Morillo CA, Verma A, Connolly SJ (2014). Radiofrequency ablation vs antiarrhythmic drugs as first-line treatment of paroxysmal atrial fibrillation (RAAFT-2): A randomized trial. JAMA.

[20] Marrouche NF, Brachmann J, Andresen D (2018). Catheter ablation for atrial fibrillation with heart failure. N. Engl. J. Med..

[21] Virk SA, Bennett RG, Chow C (2019). Catheter ablation versus medical therapy for atrial fibrillation in patients with heart failure: a meta-analysis of randomised controlled trials. Heart Lung Circ..

[22] Steffel J, Verhamme P, Potpara TS (2018). The 2018 European Heart Rhythm Association Practical Guide on the use of non-vitamin K antagonist oral anticoagulants in patients with atrial fibrillation. Eur. Heart J..

