# Author Correction: Rhythm control without catheter ablation may have benefits beyond stroke prevention in rivaroxaban-treated non-permanent atrial fibrillation

**DOI:** 10.1038/s41598-023-30971-8

**Published:** 2023-03-09

**Authors:** Wei-Ru Chiou, Po-Lin Lin, Chun-Che Huang, Jen-Yu Chuang, Lawrence Yu-Min Liu, Min-I Su, Feng-Ching Liao, Jen-Yuan Kuo, Cheng-Ting Tsai, Yih-Jer Wu, Kuang-Te Wang, Ying-Hsiang Lee

**Affiliations:** 1 Division of Cardiology, Taitung MacKay Memorial Hospital, Taitung, Taiwan; 2 Department of Medicine, MacKay Medical College, New Taipei, Taiwan; 3 Division of Cardiology, Hsinchu MacKay Memorial Hospital, Hsinchu, Taiwan; 4 Department of Biological Science and Technology, National Yang Ming Chiao Tung University, Hsinchu, Taiwan; 5 Department of Healthcare Administration, I-Shou University, Kaohsiung, Taiwan; 6 Department of Medical Education, MacKay Memorial Hospital, Taipei, Taiwan; 7 Cardiovascular Center, MacKay Memorial Hospital, 92, Zhongshan North Road Section 2, Zhongshan District, Taipei, Taiwan; 8 Department of Artificial Intelligence and Medical Application, MacKay Junior College of Medicine, Nursing, and Management, Taipei, Taiwan

Correction to: *Scientific Reports* 10.1038/s41598-022-07466-z, published online 08 March 2022

The original version of this Article contained an error in Affiliation 2, which was incorrectly given as ‘Department of Medicine, Cardiovascular Center, MacKay Memorial Hospital, New Taipei, Taiwan’. The correct affiliation is listed below:

Department of Medicine, MacKay Medical College, New Taipei, Taiwan.

The original Article has been corrected.

