# Supplementary material for: Rhythm control without catheter ablation may have benefits beyond stroke prevention in rivaroxaban-treated non-permanent atrial fibrillation
Source: Sci Rep. 2022 Mar 8;12:3745. doi: 10.1038/s41598-022-07466-z (PMC8904581; doi:10.1038/s41598-022-07466-z)
Supplement: Supplementary file 3 — Supplementary Table S1. [file 41598_2022_7466_MOESM3_ESM.docx]

Supplementary

Table S1 Multivariate Cox regression analysis for stroke (new ischemic stroke and new hemorrhagic stroke), systemic thromboembolism, HF readmission and all cause death

|  | Stroke | | | Systemic thromboembolism | | | HF readmission | | | All cause death | | |
| --- | --- | --- | --- | --- | --- | --- | --- | --- | --- | --- | --- | --- |
|  | HR | (95% CI) | P value | HR | (95% CI) | P value | HR | (95% CI) | P value | HR | (95% CI) | P value |
| Concomitant-AAD class |  |  |  |  |  |  |  |  |  |  |  |  |
| Rivaroxaban alone | 1.00 |  |  | 1.00 |  |  | 1.00 |  |  | 1.00 |  |  |
| Rivaroxaban plus AADs | 1.90 | (0.85‒4.25) | 0.119 | 0.35 | (0.14‒0.89) | 0.027 | 1.61 | (1.15‒2.25) | 0.006 | 0.73 | (0.50‒1.07) | 0.105 |
| Age (yr) | 1.04 | (0.99‒1.09) | 0.145 | 1.00 | (0.96‒1.04) | 0.988 | 1.01 | (0.99‒1.03) | 0.327 | 1.02 | (1.00‒1.05) | 0.022 |
| Female | 0.80 | (0.35‒1.84) | 0.598 | 1.05 | (0.43‒2.56) | 0.916 | 1.23 | (0.86‒1.74) | 0.254 | 1.10 | (0.73‒1.66) | 0.656 |
| Pre-CHF admission | 1.20 | (0.49‒2.94) | 0.690 | 0.54 | (0.18‒1.59) | 0.264 | 2.48 | (1.72‒3.59) | <0.001 | 1.17 | (0.76‒1.80) | 0.488 |
| Hypertension | 3.49 | (0.76‒16.07) | 0.108 | 1.35 | (0.41‒4.46) | 0.621 | 1.22 | (0.75‒1.98) | 0.415 | 0.91 | (0.53‒1.56) | 0.723 |
| Diabetes | 0.95 | (0.42‒2.18) | 0.908 | 0.89 | (0.36‒2.19) | 0.803 | 1.22 | (0.86‒1.73) | 0.259 | 1.18 | (0.77‒1.79) | 0.451 |
| Prior major bleeding | 0.49 | (0.06‒3.88) | 0.502 | 1.50 | (0.28‒8.13) | 0.637 | 1.45 | (0.78‒2.67) | 0.239 | 1.06 | (0.52‒2.17) | 0.867 |
| Prior TIA/stroke | 0.45 | (0.12‒1.61) | 0.218 | 0.31 | (0.06‒1.49) | 0.143 | 1.30 | (0.55‒3.08) | 0.550 | 1.20 | (0.50‒2.86) | 0.682 |
| Prior TIA/stroke Thromboembolism | 7.28 | (2.15‒24.64) | 0.001 | 4.14 | (1.07‒16.02) | 0.040 | 1.13 | (0.50‒2.57) | 0.764 | 1.44 | (0.62‒3.35) | 0.401 |
| Liver cirrhosis | ‒ | ‒ |  | 1.23 | (0.23‒6.62) | 0.809 | 1.27 | (0.63‒2.59) | 0.505 | 0.56 | (0.20‒1.55) | 0.265 |
| LVEF (%) | 1.00 | (0.96‒1.05) | 0.977 | 0.97 | (0.93‒1.01) | 0.174 | 0.97 | (0.96‒0.99) | <0.001 | 1.01 | (0.99‒1.03) | 0.510 |
| eGFR (mL/mm/1.73m^2^) | 1.00 | (0.98‒1.02) | 0.897 | 1.01 | (1.00‒1.03) | 0.123 | 0.99 | (0.98‒1.00) | 0.055 | 1.00 | (0.99‒1.01) | 0.753 |
| Medication |  |  |  |  |  |  |  |  |  |  |  |  |
| ACEI/ ARB | 0.73 | (0.31‒1.72) | 0.472 | 0.91 | (0.36‒2.34) | 0.850 | 1.16 | (0.79‒1.69) | 0.454 | 0.63 | (0.40‒0.99) | 0.044 |
| Beta-blocker | 0.97 | (0.42‒2.22) | 0.935 | 0.83 | (0.34‒2.05) | 0.689 | 1.18 | (0.83‒1.67) | 0.357 | 0.73 | (0.46‒1.17) | 0.196 |
| Statin | 2.15 | (0.95‒4.88) | 0.068 | 1.54 | (0.63‒3.75) | 0.342 | 0.85 | (0.58‒1.23) | 0.377 | 0.95 | (0.58‒1.58) | 0.850 |
| Aspirin | 0.73 | (0.08‒6.72) | 0.781 | 1.80 | (0.25‒13.24) | 0.564 | 1.29 | (0.62‒2.68) | 0.497 | 0.60 | (0.22‒1.62) | 0.310 |
| Clopidogrel or brilinta | 1.66 | (0.33‒8.37) | 0.538 | 0.76 | (0.12‒4.78) | 0.769 | 1.58 | (0.88‒2.85) | 0.129 | 1.33 | (0.54‒3.28) | 0.538 |
| NSAID | 1.25 | (0.39‒3.99) | 0.702 | 1.48 | (0.37‒5.97) | 0.586 | 0.95 | (0.57‒1.71) | 0.953 | 2.11 | (1.02‒4.35) | 0.044 |

AADs, antiarrhythmia drugs.
